# Effectiveness of the Virtual Reality System Toyra on Upper Limb Function in People with Tetraplegia: A Pilot Randomized Clinical Trial

**DOI:** 10.1155/2016/6397828

**Published:** 2016-01-18

**Authors:** I. Dimbwadyo-Terrer, A. Gil-Agudo, A. Segura-Fragoso, A. de los Reyes-Guzmán, F. Trincado-Alonso, S. Piazza, B. Polonio-López

**Affiliations:** ^1^Occupational Thinks Research Group, Centro Superior de Estudios Universitarios La Salle (UAM), C/La Salle 10, 28023 Madrid, Spain; ^2^Biomechanics and Technical Aids Department, National Hospital for Spinal Cord Injury, Finca la Peraleda s/n, 45071 Toledo, Spain; ^3^Health Sciences Institute, Avenida de Madrid s/n, Talavera de la Reina, 45600 Toledo, Spain; ^4^Neural Rehabilitation Group, Cajal Institute, Spanish National Research Council (CSIC), Avenida Doctor Arce 37, 28002 Madrid, Spain; ^5^Nursing, Physiotherapy and Occupational Therapy Department, University of Castilla La Mancha, Avenida Real Fábrica de Sedas s/n, Talavera de la Reina, 45600 Toledo, Spain

## Abstract

The aim of this study was to investigate the effects of a virtual reality program combined with conventional therapy in upper limb function in people with tetraplegia and to provide data about patients' satisfaction with the virtual reality system. Thirty-one people with subacute complete cervical tetraplegia participated in the study. Experimental group received 15 sessions with Toyra^®^ virtual reality system for 5 weeks, 30 minutes/day, 3 days/week in addition to conventional therapy, while control group only received conventional therapy. All patients were assessed at baseline, after intervention, and at three-month follow-up with a battery of clinical, functional, and satisfaction scales. Control group showed significant improvements in the manual muscle test (*p* = 0,043, partial *η* ^2^ = 0,22) in the follow-up evaluation. Both groups demonstrated clinical, but nonsignificant, changes to their arm function in 4 of the 5 scales used. All patients showed a high level of satisfaction with the virtual reality system. This study showed that virtual reality added to conventional therapy produces similar results in upper limb function compared to only conventional therapy. Moreover, the gaming aspects incorporated in conventional rehabilitation appear to produce high motivation during execution of the assigned tasks. This trial is registered with EudraCT number 2015-002157-35.

## 1. Introduction

The worldwide incidence of spinal cord injury (SCI) lies between 10.4 and 83 per million inhabitants per year [1]. One-third of patients with SCI are reported to have tetraplegia and 50% of patients with SCI to have a complete lesion. Related to Spain, incidence varies among 12.1 [2] and 13.1 [3], with a mean age at the time of injury of 41.8 years, and a male/female ratio of 2.6. Prevalence of SCI is estimated in 350–380 cases per million of inhabitants [2], with more frequency at the thoracic level (42.7%), following cervical (38.5%) and lumbosacral (17.8%). Most of the patients with SCI develop at least one clinical complication in their life, the most common being the loss of muscular control, sensibility, and autonomy function below the level of injury [4]. It has been estimated that all SCI discharges would need at least 4 hours a day of specialized care (occupational therapy, speech therapy, psychotherapy, physiotherapy, nursing, etc.), and around 84% of people with tetraplegia will need external help for performing activities of daily living (ADL) [5]. These needs are only a hint of the enormously devastating physical, social, and emotional burdens that individuals and their families face after a SCI [6].

Impairment of upper limbs (UL) is one of the most common sequels following neurological lesions [7], the loss of arm and hand function being one of the most devastating consequences in tetraplegia [1, 8]. In contrast with the lower limb, the UL has extensive functionality due to the mobility of numerous joints that can execute fine movements thanks to complex neuromuscular control [9]. It has been shown that most people with tetraplegia prefer the recovery of hand function to that of the bladder, bowel, or even sexual function [10]. Small progresses in arm and hand function may lead to increased autonomy in daily activities, improving independence and quality of life [11]. For this reason, improvement in UL function after cervical SCI is a top priority in individuals with tetraplegia [12].

In rehabilitation, considerable amounts of practice are required to induce neuroplastic changes and functional recovery of neurological motor deficits [13]. However, conventional therapies (CT; physical and occupational therapy) do not provide sufficient intensity for optimizing neuroplasticity because of practical limitations such as its time-consuming and labor-intensive nature, difficulty in transportation to special facilities, and need for insurance coverage [14–16]. Furthermore, traditional interventions that require simple and repetitive movements may cause monotony and boredom to the patients, reducing motivation for sustaining treatment [13, 17].

One proposed method to improve rehabilitation is to complement conventional therapy with the use of virtual reality (VR) [13]. VR is defined as a simulation of a real environment generated by computer in which, through a man-machine interface, it allows the user to interact with certain elements inside a simulated scenario [18]. VR has many advantages for intervention, such as enabling the grading of activities, obtaining precise performance measures, providing a safe and ecologically valid environment, and being enjoyable and motivating [13, 19]. In addition, VR can increase the intensity of the exercise through repetition of exercises, necessary for inducing neuroplasticity [17].

Furthermore, in spite of the many techniques available to facilitate therapy, motivation and access are major obstacles to patients in achieving the necessary dosage of movements needed for recovery [16]. Motivation can be defined as a psychological property that encourages a person's action toward a goal by eliciting and/or sustaining goal-directed behavior [20, 21] and has been showed to be a key factor for rehabilitation success [22]. The motivation of the subject with the use of VR systems is achieved by making the treatment sessions much more pleasant and attractive than conventional rehabilitation [18, 23]. Combining motivational enhancement treatments with conventional physical therapy has been shown to bring about increases in compliance with exercises [23].

While a number of VR systems have been used and have shown promising results in patients with stroke [13, 24, 25], as far as we know, experiences with people with SCI are scarce. Moreover, there is the need to further understand the relation between the detailed characteristics of these systems and the impact on the recovery of the users [26].

Toyra is a VR rehabilitation system for UL rehabilitation with people with tetraplegia. It records and reproduces in real time the movements of the patient through a personalized avatar displayed on an LCD screen [27]. The VR interface displays several common objects, where the patient is asked to reproduce the movements necessary to perform ADL. Toyra system allows also assessing UL movements by recording kinematic variables for the different degrees of freedom during the execution of analytical movements of the UL. Prior pilot studies have shown correlations between kinematic data measured with Toyra system and functional scales [28], as well as trends of improvements in kinematic, functional, and clinical variables after the ADL-based VR rehabilitation treatment [27], in people with tetraplegia. Moreover, kinematic metrics have been defined, based on data recorded by the VR system Toyra, showing promising results in terms of clinically relevant information [29].

In this paper we will discuss the hypothesis that VR combined with CT will be more effective in improving UL function than CT alone.

The aim of this pilot randomized controlled trial was (i) to investigate the effects of CT combined with an intensive and repetitive VR program on UL function in people with subacute complete tetraplegia compared with only conventional therapy and (ii) to study the satisfaction of patients with the VR system as rehabilitation supplement.

## 2. Material and Methods

### 2.1. Participants' Description

The study included 16 experimental subjects (10 males and 6 female) with motor complete cervical SCI, 11 ASIA A and 5 ASIA B [30], aged between 24 and 62 years, with an injury level between the 5th cervical and the 8th cervical vertebrae, and a mean of 4,31 ± 2,06 months after injury, and 15 control subjects (12 males and 3 female) with motor complete cervical SCI, 10 ASIA A and 5 ASIA B, aged between 19 and 65 years, with an injury level between the 5th cervical and the 8th cervical vertebrae, and a mean of 5,60 ± 2,50 months after injury (Table 1). Eligible participants met the following criteria: (1) at least 18 years of age; (2) less than 12 months from the injury; and (3) motor complete spinal cord injury according to the ASIA's impairment scale at the level of C5 to C8 (A-B ASIA level). The exclusion criteria included (1) history of traumatic or cognitive pathology that can affect the UL movements, (2) technology addiction, (3) epilepsy, and/or (4) pregnancy. All subjects presented normal or corrected-to-normal vision and hearing and tolerated the immersive VR training successfully.

After the enrolment informed consent was obtained, the 31 selected patients were assigned to 2 groups according to a simple randomization technique using sequentially numbered, opaque sealed envelopes. The envelopes containing the paper sheet with the type of treatment and a sheet of carbon paper were obscured with aluminum foil, shuffled. Then a computer generated random number list was created and a research assistant numbered the opaque sealed envelopes and placed them in a plastic container, in numerical order, to use for the allocation.

The subjects were unaware of the outcome variables considered in the study, and examiners were unaware of the experimental group assignment.

The study was carried out in the Biomechanics and Technical Aids Department of the National Spinal Cord Injury Hospital in Toledo (Spain), although the system was installed in the Occupational Therapy Department of the hospital to facilitate patients' access. All subjects were recruited from the hospital.

### 2.2. Ethics Statement

All experimental subjects signed consent forms voluntarily. The protocol conformed to the Declaration of Helsinki and was approved by the Clinical Ethics Committee of the Hospital Complex of Toledo (Spain) (CEIC 48/06-2012). Each participant received oral and writing information about experiment's details. Written consent was obtained from people able to write. When participant could not sign the consent, fingerprint was used and witness consent was signed by hospital staff or patients' relatives.

To carry out a clinical study in the hospital, the Local Ethics Committee has to check and approve the protocol of all studies. For that reason the present study was not registered before enrolment of participants started. The authors confirm that all ongoing and related trials for this intervention are registered.

### 2.3. Device Description

#### 2.3.1. Virtual Reality System

During the experimental protocol, participants were seated in their own wheelchair. The VR intervention was conducted using the VR system Toyra. This VR system consists of a television monitor and a set of inertial sensors Xsens (Xsens Inc., Netherlands) to motion capture, and scenes displayed on an LCD screen [27]. The inertial sensors captured body movements, and the subject then became immersed in the VR scene, interacting with virtual environments and objects. The captured inertial sensor data and UL anthropometric data were used to develop a biomechanical model that has been previously reported [31]. This is a wireless system so subjects moved freely in the real world while manipulating virtual objects in the 3D virtual world. Virtual sessions were designed along therapeutic guidelines for SCI interdisciplinary rehabilitation. The system offered visual and auditory feedback during the sessions, to increase the engagement, facilitate the comprehension of the exercises, and deliver a clear sense of progress.

#### 2.3.2. Training Task

In this study, one ADL-based VR game to induce UL motor skills was used. The main objective of this game was to achieve the maximum degree of autonomy that is possible in basic ADL. The monitor displayed several daily objects (spoon, fork, comb, or sponge), asking the patient to reproduce the movements necessary to perform the corresponding activities (eating, combing hair, or washing the face). The user was able to choose the preferred avatar, which represented his/her movements in mirror view in real time. Mirror view has shown to add realism and sense of presence to the practice, as well as feedback about a person's body posture and quality of movement [32]. The session offered three different difficulty levels, based on changes in objects' size and height, and speed of appearance. Subjects performed the task with their dominant arm (the arm used to perform the basic daily living tasks in the real word). If there were doubts, Edinburgh Inventory was used to established dominance [33].

### 2.4. Outcome Measures

Neurological examinations of all the patients were performed according to the ASIA standards [30]. The right and left motor indexes were determined by the manual muscle test (MMT) [34] of C5 and T1 segments from right and left extremities, respectively.

The functional examination was done by using four scales. The Functional Independence Measure (FIM) consists of 18 items organized in six categories, four corresponding to motor functions (self-care items, sphincter control, mobility items, and locomotion) and two corresponding to cognitive functions (communication, psychosocial, and cognitive). The lowest and highest scores of the total ranged from 18 to 126 [35]. The second scale was The Spinal Cord Injury Independence Measure (SCIM III) that has 16 items divided into three functional areas: self-care, respiration and sphincter management, and mobility. Total score can vary from 0 (minimal) to 100 (maximal) [36]. Only the self-care subscore has been considered in this study, because it has been previously shown that the self-care category of the SCIM III and several of its items correlate well with UL strength and capacity tests in persons with tetraplegia [37]. The Barthel Index (BI) consists of 10 items: eating, bathing, grooming, dressing, bowels, bladder, toilet use, transfers (bed to chair and back), mobility (on level surfaces), and stairs. Total score is from 0 to 100 [38]. The fourth assessment scale was the UL part of Motricity Index (MI) which assesses power and range of active movement rated for shoulder abduction, elbow flexion, and pinch between the thumb and index finger. Each movement is rated on a 0–100 point scale [39].

Clinical and functional results were analyzed based on the minimal clinically important difference (MCID) defined as “the smallest difference in score in the domain of interest which patients perceive as beneficial and which would mandate, in the absence of trouble some side effects and excessive cost, a change in the patient's management” [40], or “the smallest difference in a score that is considered worthwhile or important” [41]. In SCIM self-care subscore, approximately 1.06 to 1.22 points are necessary for MCID and 2.65 to 3.05 points for substantial meaningful changes [42], while there are 22 points in total FIM score [43]. MCID of the BI was estimated to be 1.85 points in stroke patients [44]. In the literature, no estimates were found of MCID for MMT and MI. On the basis of clinical experience and estimates reported for similar outcome measures in different domains, the MCID was set at 10% of the total range of the scales [45]. Based on that, the MCID for the MMT was 2,5 points and for the MI 10 points.

In addition to make a technical assessment of the systems, it is also important to implement a functional evaluation procedure designed by experts where the users' opinion and degree of satisfaction are taken into account. The usability concept is closely linked to the user's degree of satisfaction with the product. This concept is used to measure how useful the product and the system settings are for the user to achieve specific goals efficiently, effectively, and satisfactorily in a specific context [46].

To estimate the acceptation and motivation with the VR system as rehabilitation supplement we used the Quebec User Evaluation of Satisfaction with Assistive Technology 2.0 (QUEST) and a satisfaction survey. These tests were only evaluated in the IG, since CG did not use the VR system.

QUEST is an instrument specifically designed to measure satisfaction with a broad range of assistive technology devices in a structured and standardized way [47]. This test was designed to evaluate a person's satisfaction with his or her assistive device and can be used with adolescents, adults, and elderly people who have acquired an assistive device because of physical or sensory impairments [48]. The test includes 12 items, related to device characteristics (*n* = 8) and assistive technology services (*n* = 4). The scoring method rated from 1 (not satisfied at all) to 5 (very satisfied). Only the items related to device characteristics were used for this study, due to the lack of external assistive services. Therefore, the maximum possible score was 5 for each item, and 35 for the total scale.

We also used a satisfaction survey, to identify rehabilitation and functional aspects related to the VR system. This was a Likert scale with 20 items rated from 0 (not satisfied at all) to 5 (very satisfied), including questions about systems features, VR activities, and motivation. The maximum possible score was 5 for each item, and 100 for the total scale.

We have access to a target population in the hospital of approximately 100 cervical SCI per year, of which around 30–40% have complete injuries. Based on that, we determined that 20% of the total sample could be representative of the population. Furthermore, we have to highlight the scarce number of papers published with motor complete cervical SCI in this field of work, so we considered our sample a good representation of the target population.

### 2.5. Study Protocol

**Figure 1:**
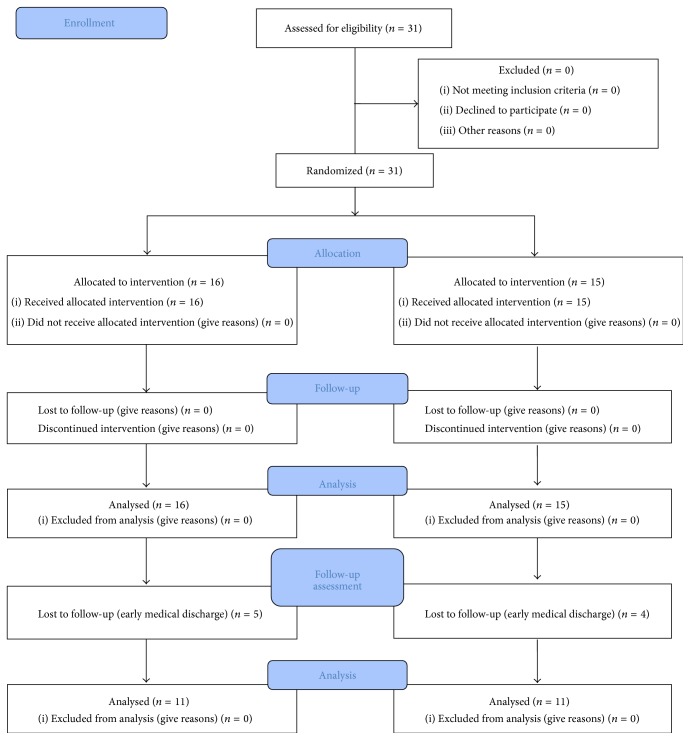
The flowchart of the trial according to the CONSORT statement. (CONSORT transparent reporting of trails, CONSORT 2010 Flow Diagram).

**Figure 2:**
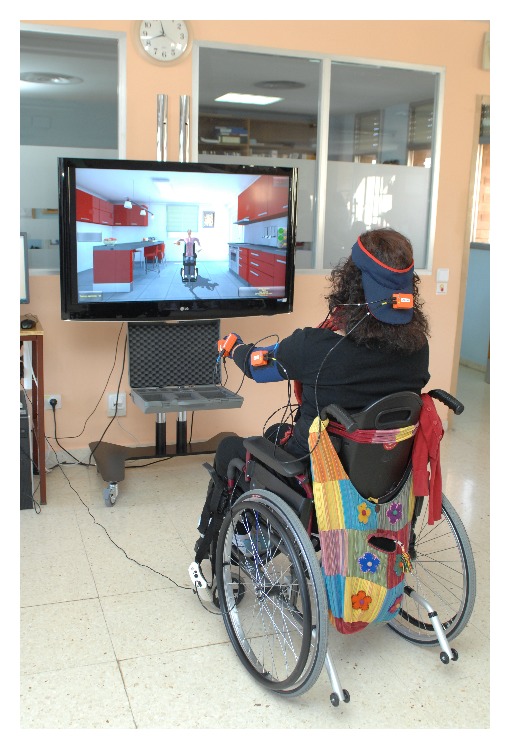
Virtual reality system Toyra. Arm movements are captured by Inertial Sensors Xsens (Xsens Inc., Netherlands). An avatar represents in real time and mirror view the movements of the user performing a task in the virtual scenario.

The experimental (EG) and control (CG) groups underwent the same CT, which consisted of routine occupational therapy and physiotherapy such as active and passive mobilizations, strengthening exercise of UL, and ADL training. CT was provided for 1 hour and 30 min per day, 5 days per week. EG received 15 sessions with Toyra system for 5 weeks, 30 minutes/day, 3 days/week in addition to CT (Figure 1). VR intervention was conducted by one occupational therapist who was professionally familiarized with VR intervention. CG made only CT (Figure 2).

The date range for participants' recruitment was from January 2011 to August 2014. Thirty-one patients (16 for EG and 15 for CG) were assessed before and after interventions. In order to know the stability of the changes, a follow-up assessment was done in 11 subjects of each group after 3 months from the VR intervention. The date range for the follow-up assessment was from February 2013 to November 2014. The other patients could not participate due to early clinical discharge.

There were no losses or discontinuity during the 5 weeks of study, nor reports of motion sickness or vertigo induced by the use of the VR system nor muscular pain during the sessions.

### 2.6. Data Collection

Every subject (CG and EG) was evaluated twice: at the beginning of the study and at the end, using a set of clinical and functional scales. The evaluation was performed the day before starting the VR treatment (preassessment) and the day after finishing (postassessment). A sample from each group (11 subjects in each group) was followed up and assessed 3 months after the study (follow-up assessment), to measure the stability of the changes. In this period both groups continued with the CT, but VR was not applied. The other persons had to give up the study due to early medical discharge, failing to complete the final assessment. The clinical and functional assessment was conducted by an occupational therapist different to the one who conducted the VR intervention and familiarized with the scales. The level of satisfaction was assessed after VR training to EG subjects.

### 2.7. Data Analysis and Statistics

The statistical analyses were conducted using SPSS 17.0 for Windows (SPSS Inc., Chicago, IL).

The independent variables were forms of intervention (CT, CT + VR therapy). The dependent variables included the UL functioning (MMT, FIM, SCIM self-care subscore, BI, and MI) and satisfaction with VR system (QUEST and a satisfaction survey). Descriptive statistics were used to analyze clinical and demographic characteristics of the subjects.

The outcome data did not deviate significantly from normality according to the Kolmogorov-Smirnov test. To compare the mean differences between groups a mixed ANOVA was used. Paired *t*-tests were performed to compare pre- and postinterventions means changes. The mean of the differences between postassessment-preassessment and follow-up-preassessments and the 95% confidence level (CI) in the dependent variables for each group was also calculated. The effect size of interventions was estimated with partial eta squared (*η* ^2^) for all dependent variables [49] where 0.20 is considered a small effect, 0.50 corresponds to medium effect, and ≥0.80 is a large effect size. It was not necessary to adjust confusion factors due to nonsignificant differences between CG and EG.

Clinical significance of the outcomes measured was assessed on the basis of the MCID defined previously.

## 3. Results

**Figure 3:**
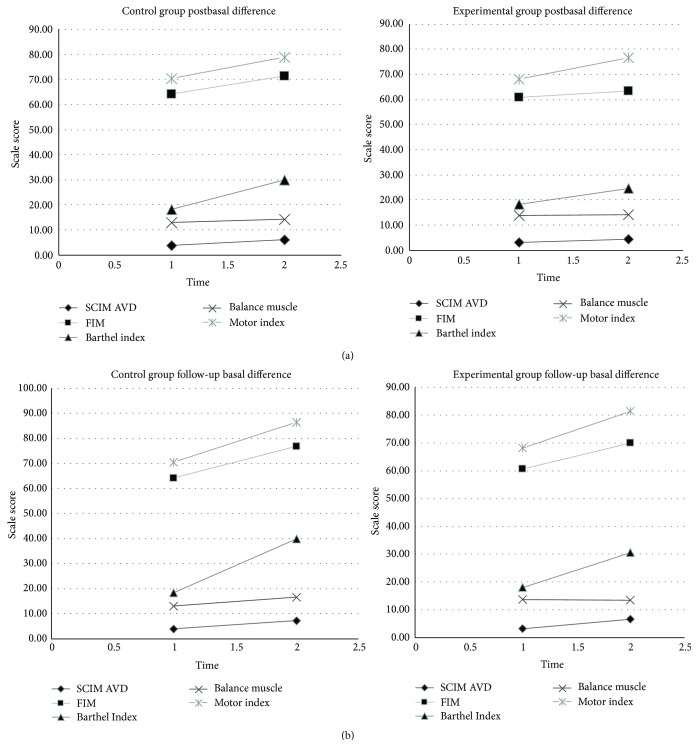
The graphs showed the tendency of the variables over the time in both groups.

We found that initial functional status was similar between groups, because no differences were found in any of the analyzed variables obtained in the battery of scales at the beginning of the study. To compare the difference in pre- and posttest scores in the EG and CG, an independent *t*-test was conducted. No group showed statistically significant improvements between pre- and posttest in the clinical and functional variables assessed. No significant differences were found between the preevaluation and follow-up evaluation. To study the improvement in UL functioning of the EG and CG, a mixed ANOVA was conducted. The CG obtained statistically significant improvements between preassessment and follow-up assessment in MMT (*p* = 0,043, partial *η* ^2^ = 0,22) (Tables 2 and 3) (Figures 3(a) and 3(b)).

In the QUEST questionnaire, the VR system obtained an overall average score of 33,20 ± 2,177 over 35 points (Table 4). The satisfaction survey scored an overall average value of 84,80 ± 8,80 over 100 points in the general satisfaction the VR system, the highest scored items being security (5 ± 0), weight, and easy adjustment (4,87 ± 0,352) with the system. Conversely, the lowest scored items were related with the similarity of the system to daily living activities (2,93 ± 1,792) (Table 5).

## 4. Discussion

The aim of this pilot randomized controlled study was to investigate the effects of CT combined with an intensive and repetitive VR program on UL function in people with subacute complete tetraplegia. The results showed that VR added to CT produced similar functional changes in UL performance than only CT. However, a high level of satisfaction of patients using the VR system was showed.

In a previous pilot study of our group, with a smaller sample and the same number of sessions, we found trends indicating improvements in functional and clinical variables after both VR and CT [27]. No significant differences were found between the two treatments in the outcome measures in this study. The literature has reported that in short intervention periods it is not possible to definitively advocate virtual reality-mediated therapy over conventional therapy for the rehabilitation of the UL [50]. Based on that, we think that the length of the interventions in this study might explain that lack of difference between groups. Also, the inclusion of other variables (e.g., kinematic) will increase the strength of our work and reinforce the utility of VR both to study and to treat motor disorders. Furthermore, it is has been showed that the level and completeness of SCI had greater influence on patients' independence in early rehabilitation period [51], since the expected level in cervical motor complete SCI was achieved by 33,3–100% of patients comparing with 87,5–100% in incomplete injuries. For that reason, big functional changes was not expected in the patients of this study, due to all sample presented motor complete injuries with several UL function limitations. To determine the influence of the injury in the effectiveness of VR treatments, it will be meaningful to include a group of people with cervical incomplete SCI in future studies.

Measurement and interpretation of functional changes guide clinical management and are primary measures of outcome [43]. It is important, then, to determine when a change in function constitutes a clinically important change. Treatment effects are typically inferred based on statistical comparisons between mean changes that result from the treatments [42]. However, a statistically significant difference does not necessarily mean a clinically important difference, which is more meaningful for both clients and clinicians [44]. One way to determine when a change in function constitutes a clinically important change is to calculate the minimal clinically important difference (MCID) for the measurement instrument [43]. In interpreting the meaning measures, it is important to consider that although small changes may be statistically significant, they may not be clinically important [41].

Both EG and CG presented MCID in SCIM self-care subscore after interventions (EG = 1,13; CG = 2,20) and substantial meaningful changes in the followed-up monitoring (EG = 3,39; CG = 3,49). These results might indicate clinical positive changes after treatments and the preservation of these changes over the time. SCIM is a disability scale developed specifically for SCI persons and has been showed to be the most sensitive to changes in function during the rehabilitation of those participants [52]. The MCID found after intervention observed in both EG (12,32) and CG (21,67) in the scale BI could indicate that patients in both groups might have clinical improvements in independency after each treatment, since the MCID of the BI in stroke patients was estimated to be 1.85 points [44]. Indeed, BI level of dependency was as follows: 0–<20 (total dependency), 20–35 (serious dependency), 40–55 (moderate dependency), ≥60 (minor dependency), and 100 (total independence). BI appeared to be a reliable test to measure ADL independence in SCI (Cronbach *α*: 0,87), existing strong correlation (Spearman-correlation: 0,69; *p* < 0,0001) between levels of injury of people with complete SCI and BI scores [52].

This is in line with results in previous results in stroke [50] where MCID were found in functional tests after both conventional VR-based treatments. The authors underline the necessity to carry out larger trials with bigger samples to reach strongest conclusions on effectiveness of VR as rehabilitation complement.

To date, research of the applications of VR in the rehabilitation of SCI is quite limited [53]. Szturm et al. [54] described a SCI case report using an interactive gaming system, coupled with the manipulation of common objects, as a form of repetitive, task-specific movement therapy. The subject after training was able to fully extend his fingers and to grasp most objects. In another study, Kizony et al. [53] demonstrated the potential of using the GestureTek's Gesture Xtreme VR system to assess static balance in 13 participants with paraplegia, showing positive responses to the experience as well as the expressions of interest in having additional sessions with the system. Ohnishi et al. [55] used isometric training with biofeedback of visual effects as in video games with a system comprising a PC and a joystick type controller comparing one person with SCI with a nondisabled participant. Results of the pilot test showed that the system is capable of assessing the differences of the individuals.

The use of computer games associated with ADLs combined with functional electrical stimulation (FES) has showed statistically and clinically improvements in hand function in people with tetraplegia [56]. However, while improvements in UL function has been showed after VR treatments in addition of CT in stroke in acute and chronic patients [14, 26, 57], the evidence of its application in tetraplegia is still very scarce. For that reason, we consider that our study adds to the limited evidence base for VR-based therapy for the UL in people with tetraplegia.

Additionally, CG obtained statistically significant differences in MMT in the follow-up assessment (*p* = 0,043; *η* ^2^ = 0,22). However, evidence indicates that MMT may not be sensitive enough to distinguish between increments at higher levels of strength or to detect the small or moderate increases seen over the course of rehabilitation in patients with SCI, because the force of a muscle reaching only 50% of the normal value, measured by objective techniques, could be rated as normal by MTT [58]. Along these lines, MI, that also measures the muscle force, showed MCID in both groups in the last assessment (EG = 13,32; CG = 15,97). It leads us to think that because these changes were found only in the follow-up assessment UL strength may have reached the peak of recovery during this period of time. It was showed that, in complete tetraplegia, almost all UL muscles with an initial strength of at least 1/5 recovered a minimum of 3/5 by 1 year [59]. Objective data from kinetic and kinematic assessment tools, for example, hand-held dynamometry or motion capture systems, would be necessary to quantify changes in patient status during the rehabilitation process.

Neither CG nor IG showed MCID after interventions in FIM scale. To this account it is important to remark that, despite being so extensively used in clinics, it has been reported that FIM scale presents strong limitations in a subpopulation of SCI [52], where the motor score is not capable of adequately discriminating the neurological level.

We think that the limited changes observed in the EG might indicate that a larger amount of time is needed for the effectiveness of the VR program to transfer to real ADL performance measured by the scales. The five-week intervention period and session's duration may not have been long enough to observe learning transfers. Furthermore, the items of the satisfaction survey related with the similarity of the system to daily living activities (2,93/5) and rehabilitation activities (1,73/5) obtained the worst scores. It leads us to think that patients did not consider VR scenario real enough to produce transferable and generalizable changes.

Patients showed great satisfaction with the VR system, all the scores in QUEST scale reaching a value higher than 3. All patients considered that the system meets their needs in terms of device's effectiveness. The satisfaction survey showed and average of 84,80 ± 8,80 in the general satisfaction with the VR system. In particular, best scored items were security (5 ± 0), weight, and ease of adjustment (4,87 ± 0,352) of the system. These results are coherent with the fact that Toyra system is based on light inertial sensors that are located with comfortable and ventilated neoprene straps, with the objective that users feel comfortable during the sessions. Furthermore, patients considered the activities proposed by the system interesting and enjoyable (4,73 ± 0,70) and felt motivated with its use (4,07 ± 1,49). Participants expressed the wish to use Toyra in their daily rehabilitation (4,33 ± 1,05) and at home (4,00 ± 2,07) and would recommend the use of the system to others (4,87 ± 0,52). Patients' motivation was shown to be an important predictor of long-term changes in quality of life and rehabilitation outcomes [60], as well as to increase the amount of time that patients are willing to spend in therapy [16].

The treatment and rehabilitation period in complete tetraplegia are long, expensive, and exhausting, because of the loss of motor, sensory, and autonomic function. Depressive disorders, psychosocial problems, and stress are frequent complications after this lesion [61]. The incorporation of VR as rehabilitation supplement has showed several personally motivating factors such as perceived control, curiosity and exploration, and imagination and socially motivating factors as cooperation, competition, and social interaction [16]. VR systems may provide more attractive and engagement treatments to people with tetraplegia, allowing users to interact with virtual objects in stimulated and variable environment, providing the user with natural control of movements using as many parts of the body as are deemed suitable within the context of therapeutic goals, and decreasing the therapist's support allowing patients to choose during their rehabilitation process (e.g., games, characters, and levels). Furthermore, the possibility to play with others can help maintain self-esteem and create positive experiences, offering opportunities for remote and proximal socialization.

For that reason, we deduce that complementing rehabilitation with a VR system might be useful to increase the dosage of therapy and to augment patient's engagement and motivation during the process.

## 5. Conclusions

This randomized controlled study showed the effects of CT combined with VR program on UL function in people with complete tetraplegia. The results showed that VR added to CT, in comparison with the only application of CT, produce similar results in UL function. However, both treatments seem to produce the minimal clinical changes necessary to consider clinically important. Moreover, the gaming aspects incorporated by VR in conventional rehabilitation appear to promote patient motivation and hence adherence to the treatment. Future research should be implemented with a larger sample size and should increase the dosage of VR therapy in terms of number of sessions.

## Figures and Tables

**Table 1:** Subject's demographic and clinical characteristics.

Variables	CG (*n* = 15)	IG (*n* = 16)
Sex (female/male)	3/12	6/10
Age [years]	40,27 ± 13,61	34,53 ± 13,71
Dominance (right/left)	8/7	12/4
Level of injury	C5(9), C6(2), C7(2), C8(2)	C5(7), C6(3), C7(5), C8(1)
ASIA (A–D)	A(10), B(5)	A(11), B(5)
Time since injury (months)	5,60 ± 2,50	4,31 ± 2,06
Etiology of damage (traumatic/postsurgical/infectious)	15/0/1	14/1/0

**Table 2:** Pre- and postassessment results of clinical and functional variables.

Variables		Preassessment		Postassessment		Postbasal difference			
		Mean	SD	Mean	SD	Mean	IC 95%	*p*	*η* ^2^
SCIM III self-care	GC	3,87	4,64	6,07	5,78	2,20	(−3,186 a −1,214)	0,118	0,082
	IG	3,25	2,30	4,38	2,47	1,13	(−2,152 a −0,098)		
	*p*	0,64		0,29					

FIM	GC	64,20	14,57	71,53	19,65	7,33	(−12,132 a −2,534)	0,066	0,112
	IG	60,69	6,78	63,25	7,26	2,56	(−5,144 a 0,019)		
	*p*	0,39		0,13					

BI	GC	18,33	16,33	30,00	22,52	11,67	(−17,739 o −5,594)	0,164	0,066
	IG	18,13	12,76	24,69	10,56	6,56	(−11,301 a −1,824)		
	*p*	0,97		0,40					

MB	GC	13,07	5,71	14,27	5,85	1,20	(−2,424 a 0,024)	0,418	0,024
	IG	13,75	5,22	14,27	5,19	0,52	(−1,577 a 0,377)		
	*p*	0,73		1					

MI	GC	70,53	18,44	78,80	20,27	8,27	(−15,998 a −0,535)	0,965	0
	IG	68,13	12,88	76,56	13,28	8,43	(−11,687 a −5,188)		
	*p*	0,68		0,72					

**Table 3:** Pre- and follow-up assessment results of clinical and functional variables.

Variables		Preassessment		Follow-up assessment		Follow-up basal difference			
		Mean	SD	Mean	SD	Mean	IC 95%	*p*	*η* ^2^
SCIM III self-care	GC	3,87	4,64	7,36	7,08	3,49	(−5,525 a −0,839)	0,944	0
	IG	3,25	2,30	6,64	7,08	3,39	(−4,893 a −1,652)		
	*p*	0,64		0,76					

FIM	GC	64,20	14,57	77,00	25,15	12,80	(−21,066 a −1,843)	0,799	0,003
	IG	60,69	6,78	69,91	12,01	9,22	(−16,866 a −3,316)		
	*p*	0,39		0,41					

BI	GC	18,33	16,33	40,00	26,17	21,67	(−30,997 a −7,185)	0,3	0,054
	IG	18,13	12,76	30,45	10,11	12,32	(−20,131 a −4,415)		
	*p*	0,97		0,27					

MB	GC	13,07	5,71	16,50	5,53	3,43	(−3,264 a −0,736)	0,043^*∗*^	0,22
	IG	13,75	5,22	13,36	5,53	-0,39	(−1,465 a 0,374)		
	*p*	0,73		0,18					

MI	GC	70,53	18,44	86,50	14,83	15,97	(−26,054 a 1,054)	0,884	0,001
	IG	68,13	12,88	81,45	12,05	13,32	(−21,535 a −5,374)		
	*p*	0,68		0,42					

^*∗*^ *p* < 0.05.

**Table 4:** Results of Quebec User Evaluation of Satisfaction with Assistive Technology (QUEST).

How satisfied are you with these system features:	Scores
(1) The dimensions (size, height, length, width) of your assistive device?	4,80 ± 0,41
(2) The weight of your assistive device?	4,87 ± 0,35
(3) The easy in adjusting (fixing, fastening) the parts of your assistive device?	4,87 ± 0,35
(4) How safe and secure your assistive device is?	5 ± 0,00
(5) How easy it is to use your assistive device?	4,60 ± 0,91
(6) How comfortable your assistive device is?	4,80 ± 0,41
(7) How effective your assistive device is (the degree to which your device meets your needs)?	4,27 ± 0,88
Total satisfaction	33,20 ± 2,17

**Table 5:** Results of satisfaction survey.

How satisfied are you with these system features:	Scores
The usability	4,53 ± 0,74
The dimensions	4,87 ± 0,35
The weight	4,87 ± 0,35
The easy adjustment of its parts	4,87 ± 0,35
The grade of comfort	4,80 ± 0,41
The instructions and comments offered	3,60 ± 1,30
The physical and psychological effort	4,13 ± 0,92
The security	5 ± 0,00
The necessary time to start-up	4,80 ± 0,56
The necessary time to perform the sessions	4,60 ± 0,74
The activities proposed by the systems are easy to perform	4,20 ± 0,77
The activities proposed by the systems are interesting and enjoyable	4,73 ± 0,70
The activities proposed by the systems are similar to my daily living activities	2,93 ± 1,79
The activities proposed by the systems are similar to my rehabilitation activities	1,73 ± 2,02
The user considers that the system can improve his/her independence in ADL	3,73 ± 1,33
The user is motivated and accept the use of the system	4,07 ± 1,49
The user would like to use the system in his/her daily rehabilitation program	4,33 ± 1,05
The user would recommend the use of the system to others	4,87 ± 0,52
The user consider that the system could be used as at home treatment (telerehabilitation)	4,00 ± 2,07
General total score that the user gives to the system	4,13 ± 0,83
Total satisfaction	84,80 ± 8,80
